# Marine heatwaves of differing intensities lead to distinct patterns in seafloor functioning

**DOI:** 10.1098/rspb.2022.1159

**Published:** 2022-11-09

**Authors:** Laura Kauppi, Anna Villnäs

**Affiliations:** ^1^ Tvärminne Zoological Station, University of Helsinki, J.A. Palménin tie 260, FI-10900 Hanko, Finland; ^2^ Baltic Sea Centre, Stockholm University, Stockholm 114 19, Sweden

**Keywords:** global warming, biodiversity-ecosystem functioning, benthic communities, sediment biogeochemical cycling, coastal ecosystems

## Abstract

Marine heatwaves (MHWs) are increasing in frequency and intensity due to climate change. Several well-documented effects of heatwaves on community structure exist, but examples of their effect on functioning of species, communities or ecosystems remain scarce. We tested the effects of short-term, moderate and strong MHWs on macrofauna bioturbation and associated solute fluxes as examples of ecosystem functioning. We also measured macrofaunal excretion rates to assess effects of temperature on macrofauna metabolism. For this experiment, we used unmanipulated sediment cores with natural animal communities collected from a muddy location at 32 m depth in the northern Baltic Sea. Despite the mechanistic effect of bioturbation remaining unchanged between the treatments, there were significant differences in oxygen consumption, solute fluxes and excretion. Biogeochemical and biological processes were boosted by the moderate heatwave, whereas biogeochemical cycling seemed to decrease under a strong heatwave. A prolonged, moderate heatwave could possibly lead to resource depletion if primary production cannot meet the demands of benthic consumption. By contrast, decreased degradation activities under strong heatwaves could lead to a build-up of organic material and potentially hypoxia. The strong variability and the complexity of the response highlight the context dependency of these processes complicating future predictions.

## 1. Introduction

Estuarine habitats are among the most productive environments in the world. They are also among the most impacted ecosystems where multiple stressors coincide [1]. On top of local or regional stressors, such as eutrophication, changes in coastal land use and fishing pressure, ongoing anthropogenic climate change has affected ecosystems and their functioning worldwide with consequences for their provision of goods and services [2,3]. Mean temperature on land as well as in the oceans has been rising globally, and the increase has been most notable at high latitudes [4]. In addition to an increase in long-term average temperatures, the severity, intensity and frequency of marine heatwaves (MHWs) are increasing [5–9] with severe effects on biota and ecosystem functioning [3,6,10]. A MHW can be described as an anomalously warm-water event that lasts at least 5 days [5,9,11], but can vary in duration, magnitude, onset and spatial extent [11].

Temperature fundamentally affects all life on Earth and organisms have long been known to have certain temperature optima where they perform best. An increase in temperature also increases the metabolic activity and energy demand of organisms [12,13], which needs to be met with available resources [14,15]. This can lead to changes in activity and feeding choice (e.g. [16,17]). For example, Lohrer *et al*. [18] showed that warming in McMurdo Sound, Antarctica, during 2010–2017 resulted in higher sediment primary production, which increased sediment metabolism and shifted benthic community composition. In addition to direct effects on organism physiology and biology, temperature may also affect animal behaviour and performance. Several consequences of both terrestrial and MHWs have been reported during the past decade including for example, geographical range shifts, shifts in species composition, harmful algal blooms and increased mortality of species [7]. For example, the Western Australian MHW in 2011 led to a tropicalization of the temperate reef ecosystem and replacement of the dominant kelp forest with persistent seaweed turfs and a shift into temperate water by tropical species [10,19]. Following this regime shift, the kelp forests have been unable to recover [19]. The 2013–2015 ‘Blob’ in the northeast Pacific caused increased mortality of marine mammals and seabirds, very low ocean primary productivity, and an increase in warm-water copepod species in the northern Californian region creating novel species compositions [20]. The Mediterranean heatwave in 2003 led to mass mortalities of rocky benthic communities, specifically gorgonian corals [21]. Although the vulnerability of the recipient ecosystem depends on its sensitivity and capacity to adapt to changes [22], the consequences of severe or extreme heatwave events are usually more dramatic than those of moderate and strong events [5].

Ecosystem functioning encompasses all the activities of organisms in the ecosystem and entails their interaction with the environment [23], often resulting in changes in, for example, biogeochemical processes. One example of this is the remineralization of organic matter in sediments, where organic matter is broken down to its inorganic nutrient constituents to be re-used by primary producers. Another example is bioturbation, that is sediment reworking and bioirrigation activities of fauna [24], which can also be seen as an indicator of species functional type and behaviour. Species functional traits (e.g. size, mobility and feeding mode) determine their effect on the sediment matrix [25]. Bioturbation enhances diffusion of solutes and microbial remineralization processes, for example, by increasing the area of oxygenized sediment for aerobic degradation processes and by enhancing porewater exchange [26]. Microbial communities are responsible for the remineralization processes and carbon loss through sediment respiration processes [27]. Climate warming may lead to changes in macrofaunal and microbial community composition and biomass, which in turn can lead to changes in their functioning and in carbon cycling [28]. Although severe changes in the structure of biological communities have been observed in heatwave-impacted ecosystems globally, and both chemical and physiological processes are temperature dependent [2,13], there are only few studies exploring impacts of MHWs on functioning at the ecosystem level. For example, Dolbeth *et al*. [29] found a slight increase in bioturbation and release of nutrients from the sediment under an experimentally induced heatwave.

Understanding the changes taking place during heatwaves in benthic communities and their functioning is essential for predicting their contribution to carbon and nutrient turnover under future climates, where extreme events become more and more common. In order to disentangle the potential effects of MHWs on ecosystem functioning, we conducted an experiment using intact sediment cores collected from aphotic (32 m), muddy sediments in the Northern Baltic Sea. We experimentally studied macrofauna bioturbation and associated solute fluxes, and community excretion rates at *in situ* temperature (corresponding to ambient temperature conditions at the time of core retrieval) and under experimentally simulated moderate and strong heatwaves. We hypothesized that the activity of the macrofaunal and microbial community, demonstrated by oxygen consumption and bioturbation rates, would increase with increasing temperature. We also expected that the macrofauna would show increasing metabolism and signs of stress with increasing temperature (expressed, for example, as increasing oxygen consumption and excretion rates).

## 2. Material and methods

### (a) Temperature conditions in the study area

The annual mean seawater temperature at a long-term monitoring site at 32 m depth in southwestern Finland, northern Baltic Sea (59° 51 318 N 23° 15 796 E) has increased by *ca* 2°C since the beginning of 1990s [30]. At the same site the frequency of MHWs has also increased [31]. In 2018, the highest temperature ever recorded at this coastal monitoring site (Tvärminne Storfjärden) reached 20.5°C in the bottom water at the end of July [30]. To identify a MHW, we followed the categorization by Hobday *et al*. [5], and based it on the mean climatology during a 30-year baseline period (1960–1990) from the Tvärminne Storfjärden monitoring site, which is located close to our sampling site (59° 51 283 N 23° 16 883 E). Generally, sea surface temperatures are used to define heatwaves, but as this study focuses on the effects of heatwaves on seafloor processes, we used available temperature data from bottom water (1 m above the sediment surface) during our study period, August–October, to calculate our baseline. Data were obtained from pelagic monitoring programmes maintained by the Finnish Meteorological Institute and Tvärminne Zoological Station, sampled every 10 days with either a CTD cast or using a Limnos water sampler.

**Figure 1.**
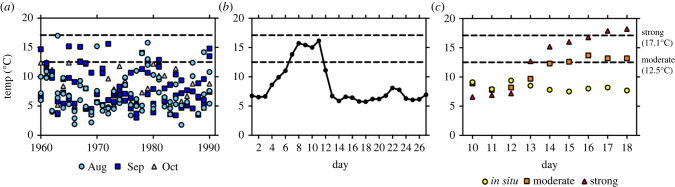
(*a*) Measured bottom water temperatures at Storfjärden during August–October 1960–1990 (30 m) (years on the *x*-axis). The 90th percentile marks the temperature defining a moderate heatwave (12.5°C), while 2 x the difference between the climatological mean and the 90th percentile defines the threshold for a strong heatwave (17.1°C; [5]). Measurements were conducted every 10 days. (*b*) Data from Storfjärden show the rapidly increasing bottom water temperature in September 2018 (days on the *x*-axis). (*c*) Observed temperature in the experimental units during the experiment. Heating started 12.9 (days on the *x*-axis). Data from the Finnish Meteorological Institute and Tvärminne Zoological Station (unpublished). (Online version in colour.)

The limit for a moderate heatwave (12.5°C) was defined as the 90th percentile of the 30-year period, while the temperature identifying a strong heatwave (17.1°C) was calculated as the local climatological mean plus twice the difference between the 90th percentile and the climatological mean ([5], figure 1*a*). The heatwave of 2018 was captured by an automated logger (figure 1*b*, MONICOAST network of loggers, logger at Storfjärden (32 m; www.helsinki.fi/monicoast)). The highly dynamic nature of this coastal area encompasses upwelling events [32] that can rapidly change bottom water temperatures, as was observed in 2018 (figure 1*b*). The temperatures in our experimental treatments corresponded to a moderate and a strong heatwave, while *in situ* temperature represents the actual bottom water temperature at the time of sampling (hereafter *in situ*, moderate and strong, figure 1*c*).

### (b) Experiment set-up

#### (i) Core retrieval and experimental design

The experiment was conducted in September 2019 at Tvärminne Zoological Station, University of Helsinki. Five intact sediment cores (inner diameter 9.0 cm) per treatment were used as experimental units and retrieved 9 September 2019, with a GEMAX twin corer (site Storfjärden). Thereafter, the experimental cores were carefully transferred to three temperature-controlled rooms, and left to settle at *in situ* temperature with continuous aeration for 3 days prior to the start of the experiment. We thereafter tested the effect of temperature increase on sediment mixing, oxygen and solute fluxes (${\mathrm{NH}}_{4}^{+}$, ${\mathrm{NO}}_{2}^{-}+{\mathrm{NO}}_{3}^{-}$, ${\mathrm{PO}}_{4}^{3-}$ and Si^4+^), bioirrigation, sediment characteristics and porewater concentrations and community excretion rates (NH_4_-N, PO_4_-P) (*N* = 5 per treatment) at *in situ* conditions and under short-term moderate and strong heatwaves (5–9 days; minimum duration of the event to be considered a heatwave is 5 days; [5]). Temperature and salinity in the experimental cores were followed daily throughout the experiment (YSI ProODO and VWR CO310 Conductivity Meter), and the water in the cores was exchanged every second day and prior to the flux measurements, using *in situ* seawater collected at the time of core sampling and stored in the treatment temperature. Water was exchanged to avoid solutes to accumulate during the experiment due to the lack of flow-through in the cores. On day 1 of the 9-day experiment, the room temperature was increased to the target temperatures (12.5–17.1°C in moderate and greater than 17.1 in strong), which allowed a gradual temperature increase in the cores mimicking a natural heatwave in this system (Figure 1*b,c*). Due to technical problems, the start temperature differed *ca* 2°C between the treatments (figure 1*c*).

#### (ii) Luminophore incubations to study sediment mixing

Concurrently with the temperature increase, luminophores were added as sediment particle tracers to study sediment particle mixing [33]. Aeration was stopped and 2.5 g DW (dry weight) of luminophores (eco-trace®, Environmental Tracing Systems Ltd, density = 2.5 g cm^−3^) were spread evenly across the sediment surface. A size fraction of luminophores corresponding to ‘mud’ with particle diameter between 10 and 70 µm was used mimicking the grain size at the sediment collection site. Luminophores were allowed to settle for 1 h before aeration was restarted. The incubation lasted 9 days. At the end of the experiment, the cores were sliced at 0.5 cm intervals down to 2 cm, at 1.0 cm intervals down to 5 cm and at 2.0 cm intervals down to 13 cm depth, and the slices were subsampled for luminophores. Luminophores were subsampled by carefully homogenizing the sediment slice on a Petri dish and thereafter transferring a small amount of sediment with a metal spoon or spatula into a zip lock bag. During the subsampling, any animals visible to the bare eye were removed and transferred to the excretion incubation (see below). The rest of the slice was then sieved through a 0.5 mm sieve to retain any additional animals left in the sediment. Due to resource constraints in handling the luminophore samples, the heated treatments were sliced first, while the *in situ* cores were sliced the following day. Sediment samples were stored in the freezer until analysis.

Sediment subsamples were freeze-dried and 0.5 g of dry sediment photographed under UV light. Luminophore pixels were counted after a binarization step (based on the RGB level) for each image corresponding to a single slice. The relative concentrations of luminophores in each slice were then used to compute the corresponding vertical depth profiles. From these, the maximum penetration depth (MPD; cm) of the tracers, and a single normal biodiffusion coefficient ($D_{b}^{N}$ in cm^2^ yr^−1^) reflecting particle mixing intensity were derived by fitting of a continuous time random walk model [34].

#### (iii) Dark incubations of solute fluxes

In the middle of the heatwaves (Day 6), dark incubations for oxygen and solute fluxes were conducted. The average incubation time for *in situ* was 3.84 ± 0.05 h, for moderate 3.03 ± 0.07 h and for the strong treatment 2.97 ± 0.06 h. We decreased the time for the heated treatments to avoid oxygen levels dropping below 80%, which would have affected the biogeochemical processes (e.g. [35,36]). Start samples were taken from the overlying water in each core and the cores were sealed with a lid with an inbuilt magnetic stirrer. At the end of the incubation, end samples were taken from the overlying water for flux calculations. Nutrient samples were filtered through a GF/F filter and frozen for later analysis, while oxygen samples were analysed by Winkler titration. Fluxes were calculated as the difference between end and start concentration of oxygen and/or solutes, and corrected for incubation time, water volume and surface area of the experimental core to represent a flux per square metre per day (24 h).

### (c) Bromide incubations to study bioirrigation

After the flux incubations, an inert bromide tracer was added to the water column of the cores to study bioirrigation. Bromide concentration in the overlying water was thereafter sampled at 0, 6, 12, 24 and 36 h. Bioirrigation was quantified in each core through the measurement of the decrease of an inert bromide (Br^–^) solute tracer spread in the overlying water. A known volume of stock NaBr solution (1 M) was introduced after the incubation to an initial Br^–^ concentration of *ca* 10 mM in the overlying water. Incubation then lasted 36 h during which overlying water was stirred using gentle aeration. Samples were kept at 4°C until analysis. The concentration of Br^–^ ions in the water samples was analysed spectrophotometrically [37] and the relation between bromide concentration in the overlying water and incubation time was assessed using simple linear regression [38,39]. Bioirrigation rates were then given as a porewater exchange rate *Q* (in ml d^−1^) calculated after [38].

### (d) Sediment characteristics and porewater concentrations

To characterize sediment properties, one additional core per treatment was kept under the same conditions and sliced together with the others at 0.5 cm intervals down to 2 cm, at 1.0 cm intervals down to 5 cm and at 2.0 cm intervals down to 21 cm depth for sediment organic matter (measured as loss on ignition), particulate organic carbon (POC), particulate organic nitrogen (PON), chl *a* and phaeophytin, as well as porewater solute concentrations. These measurements were used as supporting information for interpretation of the results, and not analysed statistically. The sediment slices were transferred into zip lock bags and sealed underwater to remove all air. Thereafter, the sediment was transferred to 50 ml Falcon tubes and centrifuged for 20 min at 4500 rpm. The supernatant was carefully pipetted to sample tubes through GF/F filters, and a subsample of the sediment was frozen for later analysis of C and N and pigments. Sediment chl *a* content was extracted with acetone (90%) for 24 h and measured spectrophotometrically. Acidification was included to identify degradation products (phaeopigments) from chl *a* [40].

### (e) Community composition and excretion rates

After luminopore subsampling, the slices were sieved through 0.5 mm sieve, and all animals visible to the bare eye (the clam *Macoma balthica*, the amphipod *Monoporeia affinis*, the polychaetes *Marenzelleria* spp., and *Bylgides sarsi* and the isopod *Saduria entomon*) were transferred to beakers containing filtered seawater to study excretion (for detailed methodology see [41]). Benthic excretion rates of ammonium (NH_4_-N) and phosphate (PO_4_-P) were measured as a community rate, that is by incubating all animals per replicate core in 105 ml of filtered (combusted Whatman GF/F) seawater over 1 h. Water temperatures corresponded to each respective treatment, and the animals were kept in darkness. Predators (*Saduria entomon* and *Halicryptus spinulosus*) were incubated separately. At the end of incubation, water samples were withdrawn with a syringe (60 ml) and filtered (GF/F) to remove faeces and pseudoefaeces. The excretion rates were corrected for nutrient concentrations of control water samples without animals for each treatment, which were incubated and treated as described above. Thereafter, the water samples were frozen (−20°C) and the animals were freeze-dried to obtain their dry weights. Any macrofauna that were not included in excretion measurements were stored in 70% ethanol for later identification under microscope to determine taxa abundance and biomass. Macrofauna taxa were divided into three functional types, surface modifiers (S), biodiffusors (B) and upward–downward conveyors (UC/DC) according to Quieros *et al*. [25] for further analysis purposes.

### (f) Statistical analyses

To check for possible differences in functional group composition of macrofauna (surface modifiers S, biodiffusors B and upward–downward conveyors UC/DC; [25]) in the experimental cores, we used non-metric multi-dimensional scaling in PRIMER 7 [42]. Different functional types affect the sediment matrix differently, thus any significant differences in the functional group composition between treatments could overrun the treatment effect. Densities and biomasses were square-root transformed before analysis.

We use a permutational ANOVA (PERMANOVA) to explore differences in the single response variables between treatments. PERMANOVA is based on a distance-matrix, using Euclidean distances. The *p*-values are obtained by a permutation procedure, testing the null hypothesis by randomly shuffling the measured values between treatments [43]. A separate PERMANOVA was run for differences in each measured parameter of excretion rates (NH_4_-N and PO_4_-P), bioturbation (MPD, $D_{b}^{N}$ and *Q*) and solute fluxes (O_2_, NO_x_-N, NH_4_-N, PO_4_-P and Si) between the treatments with treatment as a fixed factor (factor levels *in situ*, moderate and strong), using PRIMER 7 PERMANOVA add-on [43]. When examining differences in excretion between treatments, community biomass was used as a covariate in the PERMANOVA analysis, as it is known to significantly affect the excretion rate [44]. Correspondingly, when testing for the differences in bioturbation metrics between treatments, the biomass of B, UC/DC and S, and the density of S were used as covariates. Densities of B and UC/DC were discarded due to high correlation with their biomass. When exploring differences in oxygen and nutrient fluxes between treatments, bioturbation parameters ($D_{b}^{N}$, MPD and bioirrigation) were added as covariates. For the NO_x_ flux, we additionally included the O_2_ and ${\mathrm{NH}}_{4}^{+}$ fluxes as covariates, as these are known to affect nitrification–denitrification processes. Similarly, when analysing differences in the ${\mathrm{NH}}_{4}^{+}$ flux between treatments, we used NO_X_ as covariate since the magnitude of ${\mathrm{NH}}_{4}^{+}$ is affected by NO_x_ reduction. The PERMDISP procedure was used for each response variable to test for homogeneity of multivariate dispersions in the data. When needed, response variables were log_10_(*x* + 1) transformed to equalize the variance.

Finally, we performed a principal component analysis (PCA) to illustrate the treatment effect on the overall fluxes of oxygen and nutrients across the sediment–water interface. This analysis was thus performed on multiple variables (O_2_, ${\mathrm{NH}}_{4}^{+}$, ${\mathrm{NO}}_{2}^{-}$, ${\mathrm{NO}}_{3}^{-}$, ${\mathrm{PO}}_{4}^{3-}$ and Si^4+^) to illustrate the multivariate response. The analysis was based on Euclidean distances, calculated on untransformed but normalized data and performed in PRIMER 7 [42].

## 3. Results

### (a) Environmental conditions

Temperature in the *in situ* treatment varied between 7.5 and 9.4°C. This is comparable to the climatological mean at the site (August–October 1960–1990; 7.8°C). After the initiation of the heatwave, the temperature in moderate varied between 9.7 and 13.7°C, and in strong between 12.7 and 18.2°C (figure 1*c*). Salinity varied between 6.2 and 6.5 and the oxygen saturation (%) between 85.3 and 98.5 in *in situ*, 83.5 and 100.3 in moderate, and between 84.9 and 99.6 in strong.

Sediment organic matter availability (averaged over the sediment layers 0–5 cm) was lowest in moderate (8.0%) compared to *in situ* (17.0%) and strong (15.2%). Sediment phaeophytin content averaged over the sediment layers was lower in strong (122.9 µg g^−1^) than in *in situ* (127.4 µg g^−1^) and moderate (129.6 µg g^−1^). Chl *a* content, on the other hand, was lowest in the *in situ* (26.2 µg g^−1^) treatment compared to moderate (27.2 µg g^−1^) and strong (27.0 µg g^−1^). The chl *a* : phaeophytin ratio, used to assess the freshness of organic matter (ratio greater than 0.5 indicates fresh organic matter; [45]) was 0.21 in *in situ* and moderate and 0.22 in strong. Averaged over the oxygenized layer of the sediment (4 cm by visual inspection of the cores), the C/N ratio indicating the freshness of the material, was 6.9 in *in situ*, 7.3 in moderate and 7.1 in strong. At the sediment surface (top 0.5 cm) where animals feed, the C/N ratio was 7.3 in *in situ*, 7.4 in moderate and 6.6 in strong.

### (b) Macrofauna

In total, seven taxa were observed in the cores (the bivalve *Macoma balthica,* the spionid polychaete *Marenzelleria arctia,* the amphipod *Monoporeia affinis,* the priapulid *Halicryptus spinulosus,* the polychaete *Bylgides sarsi,* the crustacean *Saduria entomon* and Ostracoda). These species are adapted to a wide range of temperatures, as they occur in both shallow and deeper waters. No significant, either density- or biomass-based, differences in the functional composition between the treatments were detected (electronic supplementary material, figure S1).

### (c) Sediment mixing and bioirrigation

**Figure 2.**
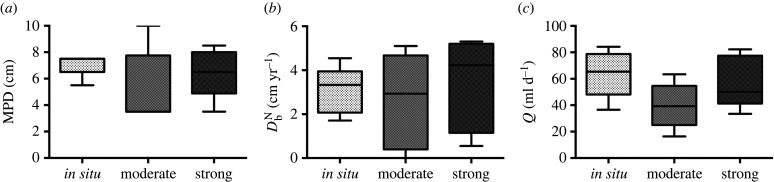
Boxplots presenting the (*a*) MPD (cm), (*b*) sediment mixing measured as the biodiffusion coefficient $D_{b}^{N}$ and (*c*) the porewater exchange rate or bioirrigation (*Q*) between the different treatments. On the *x*-axis, the *in situ* treatment represents a bottom water temperature of 7.5–9.4°C, the moderate varied between 9.7 and 13.7°C and strong 12.7–18.2°C. The boxplots mark the minimum, maximum, median and the first and third quartiles. (Online version in colour.)

The MPD (figure 2*a*) was highest in *in situ* (7.1 ± 0.9 cm, mean ± s.d. throughout) and lowest in moderate (5.2 ± 2.8 cm). The sediment mixing rate ($D_{b}^{N}$; figure 2*b*) was highest in strong (3.4 ± 2.1 cm^−2^ yr^−1^) and lowest in moderate (2.6 ± 2.2 cm^−2^ yr^−1^), whereas bioirrigation (figure 2*c*) was highest in *in situ* (63.8 ± 17.8 mL d^−1^) and lowest in moderate (39.7 ± 17.2 mL d^−1^). There were no statistically significant differences in bioturbation between the treatments (figure 2, PERMANOVA test *p* > 0.05; electronic supplementary material, table S1). The main dispersion effect (PERMDISP test) was also non-significant for all measured parameters (*p* > 0.05). The surface was completely reworked in all the cores. There was a quasi-significant treatment effect for MPD (PERMANOVA d.f. = 2, pseudo-F = 3.38, *p* = 0.09) as shown in electronic supplementary material, table S2. The biomass of biodiffusors covaried significantly with MPD (PERMANOVA d.f. = 1, pseudo-F = 13.53, *p* < 0.01).

### (d) Excretion rates

**Figure 3.**
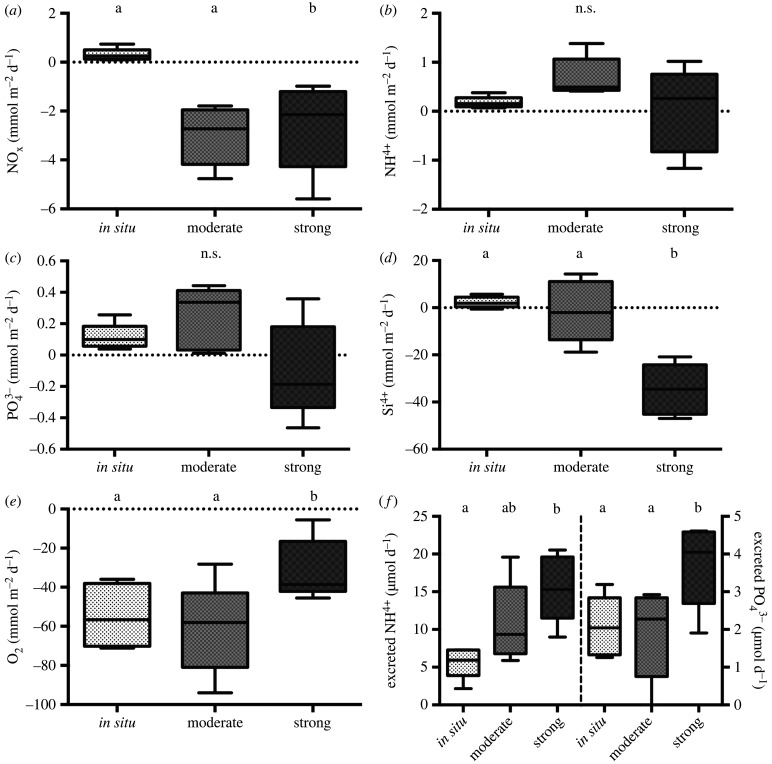
Solute (*a–d*) and oxygen (*e*) fluxes and excretion rates (*f*) in the different treatments (*in situ*, moderate and strong). The whiskers mark minimum, maximum, median, and the first and third quartiles. Different letters mark significant differences between treatments (PERMANOVA post hoc, *p* < 0.05). Note that excretion rates are represented per core (i.e. 0.006 m^2^). (Online version in colour.)

Benthic community excretion rates of NH_4_-N and PO_4_-P increased with increasing temperature (figure 3*f*) and were significantly higher in strong compared to *in situ* (PERMANOVA for NH_4_-N: d.f. = 2, pseudo-F = 7.00, *p* < 0.01 and PO_4_-P: d.f. = 2, pseudo-*F* = 6.84, *p* < 0.05; electronic supplementary material, table S2; figure 3*f*). The excretion rate of ammonium was also significantly higher in strong compared to moderate (figure 3*f*). Community biomass was a significant co-variable for NH_4_-N excretion (PERMANOVA d.f. = 1, pseudo-F = 7.24, *p* < 0.05), which increased with increasing biomass, but this relationship was not significant for PO_4_-P (PERMANOVA *p* > 0.05, electronic supplementary material, table S2). Notably, the community excretion rates were of the same magnitude as measured effluxes of ${\mathrm{NH}}_{4}^{+}$ and ${\mathrm{PO}}_{4}^{3-}$, if re-calculating values to per square metre.

### (e) Porewater and bottom water concentrations

The average porewater concentration in the first 4 cm of the sediment was higher for all solutes in moderate and strong, and for ${\mathrm{NH}}_{4}^{+}$, ${\mathrm{PO}}_{4}^{3-}$ and SiO_4_ at *in situ* conditions compared to the average concentration in the overlying water from the five flux cores per treatment. NO_X_ concentration, however, was higher in the bottom water at *in situ* conditions than in the porewater (electronic supplementary material, figure S2). ${\mathrm{NH}}_{4}^{+}$, ${\mathrm{PO}}_{4}^{3-}$ and Si^4+^ concentrations increased from top down to the deeper layers of the sediment whereas NO_x_ concentrations were highest in the first two layers of the sediment (electronic supplementary material, figure S2). Concentrations of porewater solutes were in general higher in strong compared to the other two treatments.

### (f) Solute fluxes

**Figure 4.**
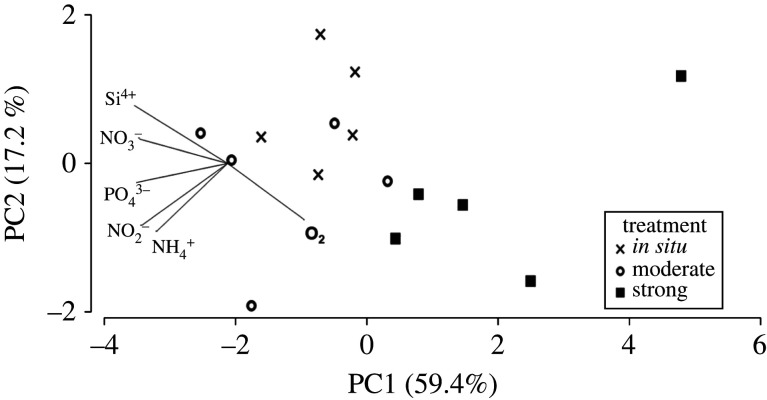
PCA of the overall fluxes of oxygen and nutrients across the sediment–water interface in each of the treatments. Oxygen consumption decreases while nutrient fluxes increase in direction of the lines. The analysis is based on Euclidean distances calculated on untransformed, normalized data.

All measured solute fluxes (NO_x_, ${\mathrm{NH}}_{4}^{+}$, ${\mathrm{PO}}_{4}^{3-}$, Si and O_2_; all results in mmol m^−2^ d^−1^) showed tendencies to differ in magnitude and direction between treatments (figure 3). Mean oxygen consumption, indicative of respiration processes, was similar in *in situ* (−54.6 ± 16.2), moderate (−61.2 ± 23.6) and lowest in strong (−31.2 ± 15.7). Similarly, the mean ${\mathrm{NH}}_{4}^{+}$ and ${\mathrm{PO}}_{4}^{3-}$ fluxes, indicative of remineralisation processes, were highest in moderate (0.7 ± 0.4 and 0.2 ± 0.2, for ${\mathrm{NH}}_{4}^{+}$ and ${\mathrm{PO}}_{4}^{3-}$, respectively) and lowest in the strong treatment (0.02 ± 0.9 and −0.1 ± 0.3 ${\mathrm{NH}}_{4}^{+}$ and ${\mathrm{PO}}_{4}^{3-}$, respectively; figure 4). Mean silicate flux varied from an efflux in the *in situ* treatment (2.3 ± 2.3) to a slight influx in moderate (−1.4 ± 13.1) and an higher influx in strong (−34.7 ± 10.8). The mean *in situ* NO_X_ flux was slightly positive (0.3 ± 0.2), and decreased and became an influx towards moderate (−3.0 ± 1.2) and strong (−2.7 ± 1.9; figure 3).

PERMANOVA test revealed a statistically significant treatment effect for NO_x_ (d.f. = 2, pseudo-F = 10.62, *p* < 0.01), Si^4+^ (d.f. = 2, pseudo-F = 16.48, *p* < 0.01) and O_2_ (d.f. = 2, pseudo-F = 11.28, *p* < 0.01; figure 3; electronic supplementary material, table S2). Pairwise comparisons showed that strong differed significantly from *in situ* and moderate (*p* < 0.05) but no significant difference was found between *in situ* and moderate (*p* > 0.05). Also ${\mathrm{PO}}_{4}^{3-}$ flux showed a similar response and the main treatment effect was close to significant (PERMANOVA d.f. = 2, pseudo-F = 4.16, *p* = 0.06). The ${\mathrm{NH}}_{4}^{+}$ flux significantly covaried with the NO_X_ flux (PERMANOVA d.f. = 1, pseudo-F = 13.47, *p* < 0.01) but did not have any significant treatment effect (*p* > 0.05). Similarly, ${\mathrm{NH}}_{4}^{+}$ (PERMANOVA d.f. = 1, pseudo-F = 10.98, *p* = 0.01) and bioirrigation (PERMANOVA d.f. = 1, pseudo-F = 6.26, *p* < 0.05) significantly covaried with NO_X._ Bioirrigation was also a significant covariate for O_2_ consumption (PERMANOVA d.f. = 1, pseudo-F = 29.12, *p* < 0.001). Overall, if considering the fluxes together, PCA clearly summarizes that oxygen consumption increases together with the nutrient fluxes towards the *in situ* and moderate treatments compared to the strong treatment (figure 4).

## 4. Discussion

As coastal ecosystems around the world are already under multiple pressures [1], including eutrophication, hypoxia and acidification, disentangling the effects of MHWs is necessary for forecasting possible future changes in marine ecosystems [22]. Here, we explored the effects of short-term moderate and strong MHWs on sediment ecosystem functioning. The remineralization of organic matter in the sediment recycles nutrients back to the primary producers, which are the basis of the food web. Proper functioning of the seafloor is therefore essential for sustaining marine ecosystems. Our results suggest significant changes in the biogeochemical cycling of nutrients within the seafloor under strong heatwaves, while moderate heatwaves seem to boost remineralisation processes. Strong heatwaves also lead to increased metabolism and stress of benthic animals, although they are able to maintain their mechanistic processes.

### (a) Heating did not decrease macrofauna bioturbation

Bioturbation performed by benthic macrofauna is an essential ecosystem process, which alters the redox conditions of the sediment matrix thereby affecting the rate of chemical processes [26]. The mechanism by which different species affect these rates depends on their functional traits. We found no effect of temperature on sediment mixing rate, MPD of luminophores (fauna) in the sediment or bioirrigation rates. The lack of significant differences in bioturbation between the treatments is not surprising given that there were no significant differences in macrofauna community (functional) structure between the experimental units. However, the general mechanistic effect of bioturbation was sustained even under strong heatwave conditions, indicating that, on a community level, the macrofauna was still able to perform its functional task. There can be inter- or even intraspecific variation on the responses of individuals to heatwaves [17,46–48], which could cause the increasing variation seen in the bioturbation parameters from *in situ* towards strong heatwave conditions, thus hampering the detection of statistically significant differences. However, the increasing variation per se already indicates an effect of heatwaves on bioturbation processes (figure 2). Depending on the temperature optima of different taxa, some species might be expected to decrease their movement and feeding activities towards higher temperature. Thus, the decrease in oxygen consumption seen in the strong treatment could be an indication of macrofauna decreasing their activity due to heat shock. This is, however, unlikely since the sediment mixing and bioirrigation remained at the same level throughout the different treatments.

### (b) Macrofauna metabolism increases with temperature

Our results showed that community excretion rates increased with temperature and biomass, although the effects of temperature were only significant under the strong heatwave treatment. Indeed, the metabolic process of excretion is predicted to increase with increasing temperatures [13,44]. The higher excretion rate indicates a higher metabolic rate of benthic species, but could also partly be interpreted as a stress response of the animals in response to the warming temperatures [41]. Handling of the animals before incubation is also likely to induce stress in the animals [41]; however, animals from all treatments were handled in a similar manner.

### (c) Heating affects sediment biogeochemical processes

**Figure 5.**
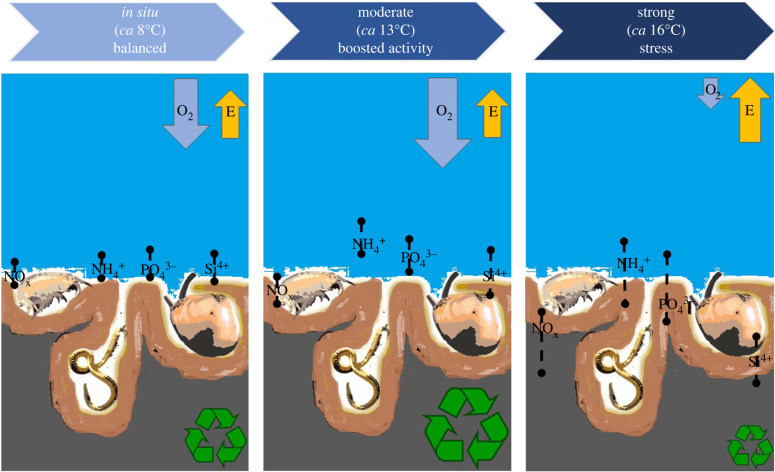
A conceptual figure summarizing and combining the results of the measured parameters. Macrofaunal communities and ecosystem functions respond differently depending on the intensity of the heatwave. Even though the mechanistic activities of macrofauna remain unchanged, metabolism (as estimated by excretion (E); yellow arrow) increased with temperature. A moderate heatwave was observed to boost oxygen consumption (O_2_, light blue arrow) and nutrient recycling processes (symbolized by the size of the circular, green arrows) in the sediment, which then decreased under a strong heatwave. Magnitude in fluxes is illustrated with their position at the sediment–water interface, whereas the length of the dashed line indicates the amount of variation in each flux. The dominant benthic species (the bivalve *Macoma balthica*, the amphipod *Monoporeia affinis* and the polychaete *Marenzelleria* spp.) and their position in the sediment is illustrated. (Online version in colour.)

Interestingly, we found differences in both magnitude and direction of sediment biogeochemical fluxes between treatments (figure 5) despite the lack of statistically significant differences in benthic bioturbation or functional composition of the macrofauna. Similarly to our results, Sanz-Lázaro *et al*. [49] found no significant effect of temperature on bioirrigation by *Nereis diversicolor,* although they found a significant effect of temperature on sediment metabolism mediated by *Nereis* bioirrigation. In this experiment, bioirrigation rates are at the same level in strong and in the *in situ* treatment. However, in the (normal) *in situ* conditions, we observed a slight efflux of solutes while in the strong heatwave conditions an influx of oxygen-associated solutes, such as nitrate-nitrite and phosphate was observed. The influx in the strong treatment could partly be due to mechanistic effects of the fauna on sediment oxygenation. Hence, the solutes associated with oxygen, such as phosphate and silicate, are trapped in the sediment. This also explains the importance of sediment mixing and bioirrigation as covariables for these fluxes. It should be noted that the fluxes in the *in situ* treatment can be somewhat overestimated compared to the heated treatments due to a slightly longer incubation time (maximum 53 min difference). However, because the fluxes are given as millimoles per square metre per day, this difference in incubation time should not affect the results. The low temperature in the *in situ* treatment did not allow a shorter incubation time based on previous studies [35,36], to get measurable results. A shorter incubation time was, however, necessary for the heated treatments to keep oxygen level saturated (greater than 80%).

Overall, the negligible solute effluxes and the oxygen consumption in *in situ* conditions are comparable to previous studies conducted in organic-rich, muddy sediments in the area (e.g. [36,50]). The increase in especially ammonium, phosphate and silicate fluxes in the moderate treatment is indicative of both a purely chemical increase in diffusion rates, increased metabolic activity of organisms in higher temperature as well as higher activity of degradation processes. Oxygen consumption of the sediment can be used as a proxy for the activity of both macrofauna and microbial processes in the sediment [51]. Hence the increase in oxygen consumption simultaneously with the aforementioned solutes in moderate indicates a boost in nutrient cycling. In the strong treatment, however, the oxygen consumption decreases almost to half of that in the two other treatments, indicating a lower activity of sediment processes. Similarly, the silicate flux, which is sensitive to movements of particles and water, while also being a degradation product of diatoms, has a large influx in the strong treatment. However, ammonium and phosphate fluxes remain rather high in the strong treatment, possibly due to the increase in metabolic activity of macrofauna (figure 5).

### (d) Indications of changes in organic matter cycling in response to heatwaves of different intensity

Even though we did not measure microbial activity in this experiment, temperature likely has a direct effect on the microbial community and thus organic matter degradation processes [52–54]. For example, soil warming by 5°C could result in a decline in microbial and degradative enzyme biomass and thereby a decrease in the carbon flow from soil to atmosphere [28]. On the other hand, thermal adaptation or change in the community structure in response to warming towards species more efficient in degradation could counteract the loss of biomass [54]. Microbial activities break down organic matter to its molecular constituents. In addition to temperature, the breakdown of organic matter into its molecular constituents by microbial activities is also much more effective under aerobic than anaerobic conditions. Sediment reworking and burrow ventilation activities by benthic macrofauna increase oxygenized surfaces where microbial remineralization of organic matter can occur. Our results show that sediment mixing and burrow ventilation rates in *in situ* and strong are similar to each other, despite the high variability in strong, and along with visual evidence, indicate that oxygen was available in the sediment for aerobic processes. However, the low oxygen consumption and the low correlation between ammonium and phosphate fluxes, as well as the lower sediment C/N ratio in strong (6.6) compared to the *in situ* (7.3) and moderate (7.4), indicate decreased microbial processes in strong heatwave conditions resulting in the freshest organic material in these treatments.

On the other hand, the highest oxygen consumption and correlation between ammonium and phosphate, the lowest organic matter and chl *a* concentration, highest phaeophytin, as well as highest C/N ratio, were observed in moderate. This is indicative of a functioning microbial community and implies the highest efficiency of organic matter degradation during a short-term, moderate heatwave. This is in line with the results of Redfern *et al*. [17] who observed the highest activity and probability of feeding at intermediate temperature, and of Baker *et al*. [55] who similarly observed high nutrient uptake rates in intermediate temperatures. By contrast, Walker *et al*. [52] found, by combining field observations and experimental work, that microbial activity is temperature-dependent and does not acclimate to a warming of 6°C over the course of weeks or even decades. Based on our bioturbation and excretion measurements as well as the evidence from solute fluxes above, macrofauna were still active in strong heatwave conditions, and thus a decrease in microbial activity most likely explains the decrease in remineralisation processes under a short-term, strong heatwave. Since we did not offer any fresh food for the macrofauna, it is also possible that the response seen in the strong treatment is due to the macrofauna shutting down their activities in response to the decreased food supply. However, results from organic matter, POC, PON and pigment analyses suggest that fresh material was available and our results from the bioturbation measurements show no changes in the activity of macrofauna.

### (e) The need to encompass complexity and variability of natural systems

This experiment was designed to explore the effects of short-term heatwaves of differing intensities on benthic ecosystem functioning. The different response variables measured the effects of heatwaves on different timescales. As we used intact sediment cores, they contained the legacy effect of the community contained in the core. The measured bioturbation rates are therefore an integration over several days and not only measured the immediate effects of heatwaves across the short term. Therefore, significant treatment effects could be difficult to detect even if there were some. Solute fluxes, excretion rates and sediment oxygen consumption represent a snapshot of the immediate effects of a heatwave and give information regarding the short-term response. Our design did not allow the study of individual species, but this could be an important aspect in future experiments. Given that different species have different thermal tolerance limits, knowing the effects of single species might help us predict the consequences for ecosystem functioning where we are facing a loss of some species while gaining others.

Due to the design of the experiment, we were not able to follow up on the possible recovery phase after the heatwave. For future predictions, it would be crucial to know if and when the ecosystem functions recover and whether or not the consequences are dependent on the intensity, length or frequencies of heatwaves. In the experiment by Amorim *et al*. [56], for example, survival and condition of the bivalve *Scrobicularia plana* were not affected by the exposure to extreme heatwaves. However, longer heatwaves with shorter recovery periods induced a physiological defense mechanism, which was normalized during recovery. Our results indicated a decrease in organic matter degradation under a strong heatwave. If this function does not recover in a reasonable time, organic matter would start to deposit on the seafloor in excess, potentially leading to hypoxia. The risk of hypoxia during heatwaves is further increased by the capacity of warmer water to retain less oxygen than colder water. Although our treatments mimicked natural conditions observed at the sampling site during the heatwave of 2018, no hypoxia was observed in the bottom water during this time. However, hypoxia and/or other stressors do co-occur with heatwaves [22,57–59] and experiments on multiple stressors are needed to reveal possible synergistic and/or antagonistic effects. There is also indication from the study site that hypoxia following a long-term heatwave leads to a degradation of the benthic community, indicating a legacy effect of the abnormally high temperature (L.K. & A.V. 2021, pers. obs.). In the study area, changes in salinity have also been expected as a result of climate change [60]. A decrease in salinity would cause further stress on the marine biota, many of which are already living at the edge of their distribution range in the brackish Baltic Sea. Coupled with heatwaves, the low salinity might push many of the marine taxa over the edge.

## 5. Conclusion

Climate change and the consequent increase in intensity and frequency of MHWs are threatening the biodiversity as well as the functioning of coastal seas. Our results suggest that macrofaunal communities and ecosystem functions respond differently depending on the intensity of the heatwave. Even though the mechanistic activities of macrofauna (sediment mixing and burrow ventilation activities) did not significantly change, a moderate heatwave seemed to boost nutrient recycling, which then decreased under a strong heatwave. Should a moderate heatwave last for a longer time, the boost in degradation processes could result in substrate depletion [52] with consequences for several aspects of the macrobenthic community [15,61]. On the other hand, the decrease in degradation processes under a strong heatwave could over time lead to a build-up of organic matter on the seafloor, which would cause oxygen depletion. For the great majority of our measured parameters, variation increased from *in situ* towards strong heatwave conditions, suggesting that the predictability of the consequences of a heatwave decreases in response to its strength, at least up to the point where the limit of thermal tolerance is reached and the system collapses. Considering the vast area of marine sedimentary ecosystems and their importance for regulating cycles of carbon and nutrients, studies in aquatic systems are sorely needed.

## Data Availability

The dataset underlying this study is available from the Dryad Digital Repository: https://doi.org/10.5061/dryad.2v6wwpzrx [62]. The data are provided in the electronic supplementary material [63].
